# Analysis of the influence of flame sterilization included in sampling operations on shake-flask cultures of microorganisms

**DOI:** 10.1038/s41598-020-66810-3

**Published:** 2020-06-30

**Authors:** Masato Takahashi, Takafumi Honzawa, Ryuichi Tominaga, Hideki Aoyagi

**Affiliations:** 1 Faculty of Life and Environmental Sciences, University of Tsukuba, Tsukuba, Ibaraki 305-8572 Japan; 2 Combustion of Thermo and Fluid Dynamics, Department of Fundamental Technology, Tokyo Gas Co. Ltd., Yokohama, Kanagawa 230-0045 Japan

**Keywords:** Applied microbiology, Bacterial techniques and applications

## Abstract

Shake-flask cultures of microorganisms involve flame sterilization during sampling, which produces combustion gas with high CO_2_ concentrations. The gaseous destination has not been deeply analyzed. Our aim was to investigate the effect of flame sterilization on the headspace of the flask and on the shake-flask culture. In this study, the headspace CO_2_ concentration was found to increase during flame sterilization ~0.5–2.0% over 5–20 s empirically using the Circulation Direct Monitoring and Sampling System. This CO_2_ accumulation was confirmed theoretically using Computational Fluid Dynamics; it was 9% topically. To evaluate the influence of CO_2_ accumulation without interference from other sampling factors, the flask gas phase formed by flame sterilization was reproduced by aseptically supplying 99.8% CO_2_ into the headspace, without sampling. We developed a unit that can be sampled *in situ* without interruption of shaking, movement to a clean bench, opening of the culture-plug, and flame sterilization. We observed that the growth behaviour of *Escherichia coli*, *Pelomonas saccharophila*, *Acetobacter pasteurianus*, and *Saccharomyces cerevisiae* was different depending on the CO_2_ aeration conditions. These results are expected to contribute to improving microbial cell culture systems.

## Introduction

Rotary shake-flask culturing was first used for the cultivation of *Aspergillus flavus* in 1933^1^, and is now widely used for the cultivation of various microorganisms. Shake-flask culturing is different from surface culturing where the cells are directly exposed to high oxygen concentrations. In shake-flask cultures, the cells are exposed to low oxygen concentrations since the microorganisms are suspended in the culture broth. Therefore, a lot of work has been done to improve oxygen supply to shake-flask cultures. For example, a shouldered, flat-bottom Kolben (termed a Sakaguchi flask) for reciprocating, shaking cultures was developed for submerged cultures of *Aspergillus*^2^. In recent years, new shaking methods using resonant acoustic mixing with ~6 times higher *k*_L_a have been reported^3^.

In the past, it was difficult to monitor various culture parameters in shake-flask cultures in both the headspace and culture broth because of the high-speed shaking. However, with recent advances in measurement technology, various monitoring devices have been developed^4^. We developed a new bypass-type device that can monitor O_2_ and CO_2_ concentrations by circulating the medium and gas in shake-flask cultures^5^. This differs from conventional monitoring devices^6–11^. A device that combines a respiration activity monitoring system^11^ (a direct type) and a bypass device have also been reported recently^12^. The Circulation Direct Monitoring and Sampling System (CDMSS) uses a method where the liquid and gas are circulated from the flask to the bypass ports for a short duration^5^. Its functions differ from those of the direct type and allow more than monitoring during shake-flask culturing^4^. We reported a culture method in which the CO_2_ in the flask gas phase is removed and dissolved CO_2_ concentration is minimized by placing a CO_2_ adsorption column into the CDMSS^5,13^. Vertical gradients in gas levels (especially CO_2_) in the flask headspace have been demonstrated under different conditions (e.g., circulation speed, measurement site)^13^. As described above, the gas concentration can be monitored in real time, such that shake-flask cultures have been observed to have a complex gas environment.

Presently, monitoring devices for shake-flask cultures are not yet common. Flask shaking is often interrupted in order to investigate changes in various culture parameters with time and the sampling procedure has no established restrictions, except for the prevention of contamination. This process is dependent on the skills of the experimenter. A temporary decrease in oxygen transfer rate has been reported when the flask is taken from the shaker to a laminar flow bench to perform sampling^11,14^. Moreover, it has been reported that, during shake-flask culturing of soil samples, different structures of microbial communities are formed depending on sampling and intermittent opening of the culture-plug for as little as 30 s^15^. Some cultivation experts may also be aware of the effects of subtle manipulations based on empirical knowledge. However, except for the above, there is currently no report on the influence of the sampling operation on shake-flask cultures. Thus, there is a paucity of information on the influence of conventional sampling procedures on culture parameters. We, therefore, investigated other possible effects of the sampling operation (other than the interruption of shaking and opening of the culture-plug) on microorganism growth.

We have reported previously that the changes in CO_2_ concentration in the flask gas phase during shaking culture affect the structure of the cultured microbial community^15^. Among the various operations in standard sampling, it is presumed that those causing changes to the gas environment of the flask (e.g., flame sterilization or opening the culture-plug) have significant effects on the microorganisms in the culture broth. However, it is difficult to evaluate the direct influence of flame sterilization and culture-plug opening on the culture, since these procedures are complicated manual operations that are accompanied by interruption of shaking. Reportedly, with computational fluid dynamics (CFD) analysis, there is a high concentration of CO_2_ around the Bunsen burner flame due to the combustion of methane^16^. Based on this information, we hypothesized that the high concentration of CO_2_ generated at the flask mouth during flame sterilization would move into the flask headspace. Therefore, we designed experimental systems to test this hypothesis. The present study combined CFD analysis of the flame sterilization operation, CDMSS^5^, and a system that can aerate the flask gas phase (termed Automatic Aeration Flask System (AAFS))^15^. The aim was to analyze the effect of flame sterilization on the headspace of the flask and on the shake-flask culture.

## Results

### CO_2_ concentration during manual flame sterilization

**Figure 1:**
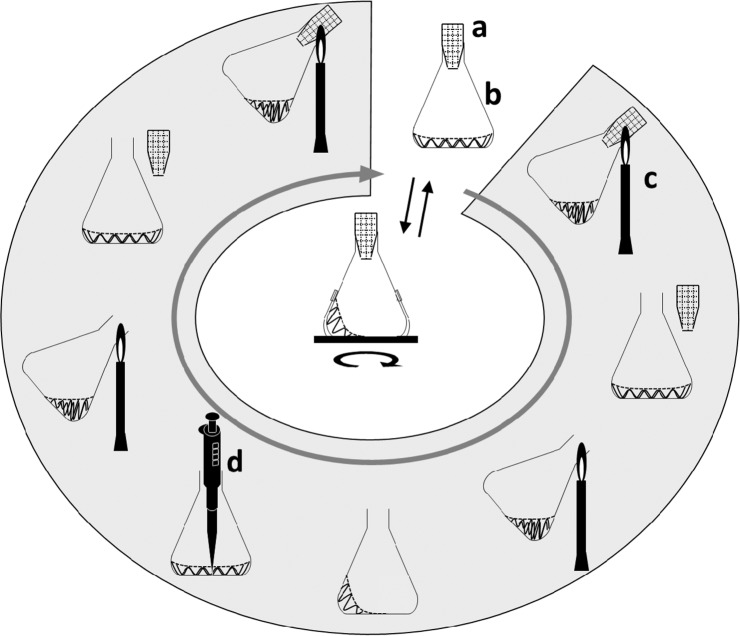
Conceptual diagram of standard sampling process in conventional shake-flask culture. Gray shading indicates operation on a clean bench. (**a)**, breathable culture-plug; (**b)**, flask; (**c)**, Bunsen burner; (**d)**, pipettor.

**Figure 2:**
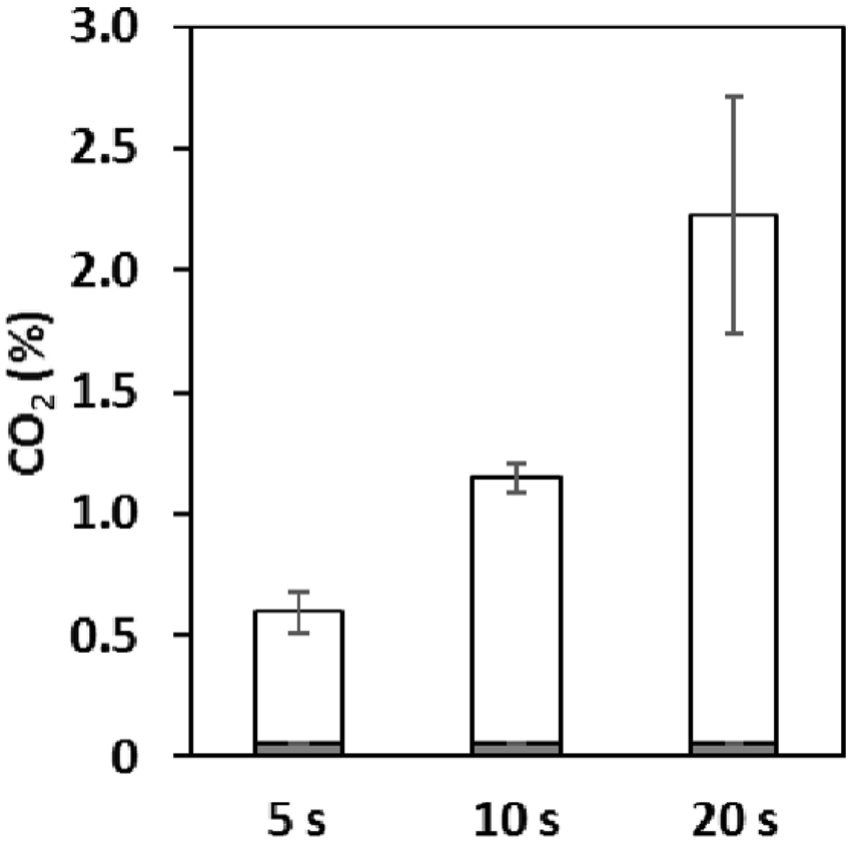
Maximum CO_2_ concentration in the headspace of flasks due to manual flame sterilization of the flask mouth. White bars, maximum concentration; Gray bars, initial concentration. Error bars indicate standard deviations (n ≥ 4).

**Figure 3:**
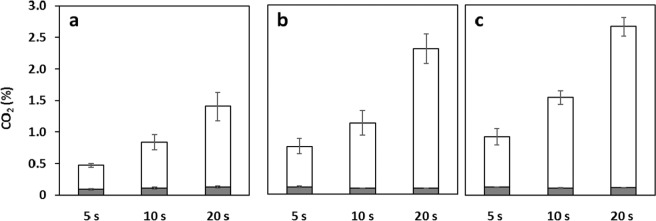
Maximum CO_2_ concentration in the flask headspace due to flame sterilization of the flask mouth using the system described in **Fig. S1** online. White bars, maximum concentration; Gray bars, initial concentration. Conditions (**a–c)** represent inclination angles (θ) of 15 °, 25 °, and 35 °, respectively. Error bars indicate standard deviation (n = 4).

The sampling of culture flasks is usually performed according to the procedure shown in Fig. 1. The influence of the flame sterilization procedure on the physiochemical properties of the flask was studied before investigating the effect of the procedure on the microorganisms. During the standard, manual sampling process, it was clear that prolongation of flame sterilization of the flask mouth readily filled the headspace of the flask with CO_2_ **(**Fig. 2**)**. During the manual flame sterilization, the CO_2_ concentration in the headspace increased from 0.50 to 2.0% (v/v) as the flame sterilization process was prolonged from 5 to 20 s. The variation increased significantly as the operation time continued because the procedure was manual **(**Fig. 2**)**. It was also observed that, using a system similar to that shown in Supplementary Fig. S1 online, the larger the inclination angle (θ value, from 15–35 °), the easier it was for the headspace of the flask to fill with CO_2_ **(**Fig. 3**)**. Thus, we have demonstrated for the first time, to the best of our knowledge, that flame sterilization during manual sampling of conventional shake-flask cultures results in CO_2_ accumulation in the headspace. At both flame sterilizations of the angle of inclination of 25 ° and the routine manual work, as time increased by 1 s, the CO_2_ concentration increased by ~0.1% (v/v) **(**Figs. 2 and 3**)**. All measurements were performed using CDMSS and the gas phase inside the flask was mixed and measured accordingly.

### CO_2_ behavior in flame sterilization

**Figure 4:**
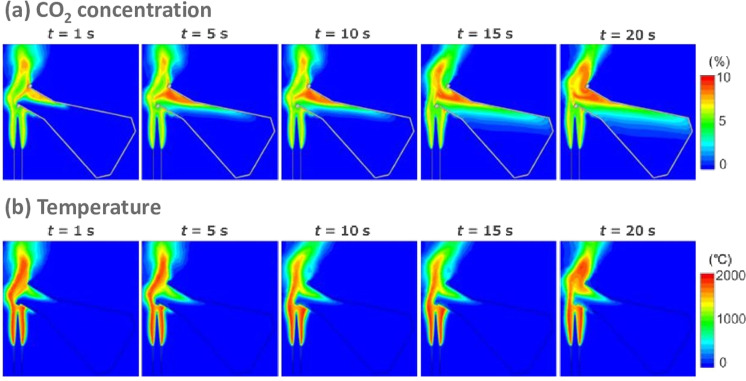
Distributions of **(a)** CO_2_ concentration and **(b)** temperature in a vertical cross section passing through the center of the flask.

A CFD analysis was also performed in order to understand, in more detail, the effect of the flame sterilization procedure. Figure 4 shows the distributions of **(a)** CO_2_ concentration and **(b)** temperature on a vertical cross section through the center of the flask from 1–20 s. The CO_2_ concentration inside the flask increased with time. The temperature exceeded 500 °C in the upper portions of the flask, but remained ambient in other portions. Near the inner wall of the flask, there were regions where the CO_2_ concentration was high and the temperature was low. This suggests that water vapor in the burned gas condensed. It should be noted that condensed water, which includes CO_2_ and nitrogen oxides and is acidic (pH ~ 3), is produced if the flask’s surface becomes foggy. Supplementary Figs. S2 and S3 online show the time-dependent changes in CO_2_ concentration and temperature along the center axis of the flask. As mentioned above, CO_2_ inflows as time passes, reaching a maximum concentration of approximately 9%, which is lower than the concentration when methane undergoes stoichiometric combustion. In contrast, the temperature remains nearly constant after *t* = 2 s, which indicates that the culture fluids would not be heated by this process.

**Figure 5:**
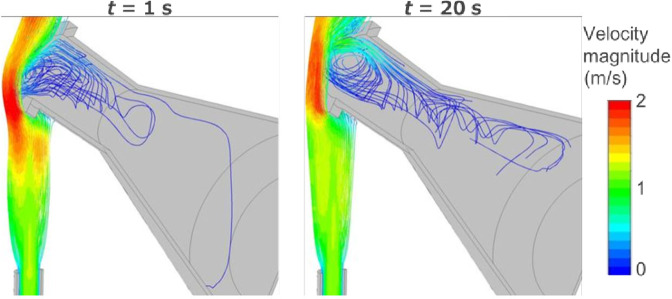
Streamlines from the burner inlet at t = 1 and 20 s.

Regarding the flow of burned gas inside the flask, Fig. 5 shows the streamlines from the burner inlet at *t* = 1 and 20 s. The midline cross section is shown for clarity and the line colors denote the magnitude of velocity. Incidentally, when the flask is placed in the flame at *t* = 0 s, it becomes a barrier to the flame’s flow. Clockwise eddies are generated downstream. The shape of the eddies essentially does not change between *t* = 1 and 20 s, but the velocity does increase. Because the burned gas possesses increased temperature and buoyancy, it inflows along the upper part of the flask. Then, the gas is cooled, and CO_2_ and H_2_O flow inside the flask by diffusion and advection.

### Effect of intermittent CO_2_ changes on microorganisms during shake-flask culture

Considering the conventional sampling process of the shake-flask culture **(**Fig. 1**)**, when investigating the influence of CO_2_ accumulation in the gas phase of the flask due to flame sterilization, the interruption of shaking and opening the culture-plug will also affect the culture. Thus, separating the effects of flame sterilization from other effects is not possible using the conventional method. Therefore, the AAFS^15^, which can introduce fresh air into the flask aseptically without interrupting the culturing, was modified as shown in Supplementary Fig. S4 online to allow intermittent aeration with 99.8% (v/v) CO_2_. At a CO_2_ aeration rate of 30 mL/min, the increase in CO_2_ concentration was the same as that obtained during flame sterilization at an inclination angle of 25 ° **(**see Supplementary Fig. S5 online**)**. The maximum CO_2_ concentration increased in proportion to aeration time. Using a 500 mL Erlenmeyer flask aerated with 99.8% (v/v) CO_2_ at 30 mL/min, as the aeration time increased by 1 s, the CO_2_ concentration increased by ~0.1% (v/v) **(**see Supplementary Fig. S5 online**)**.

**Figure 6:**
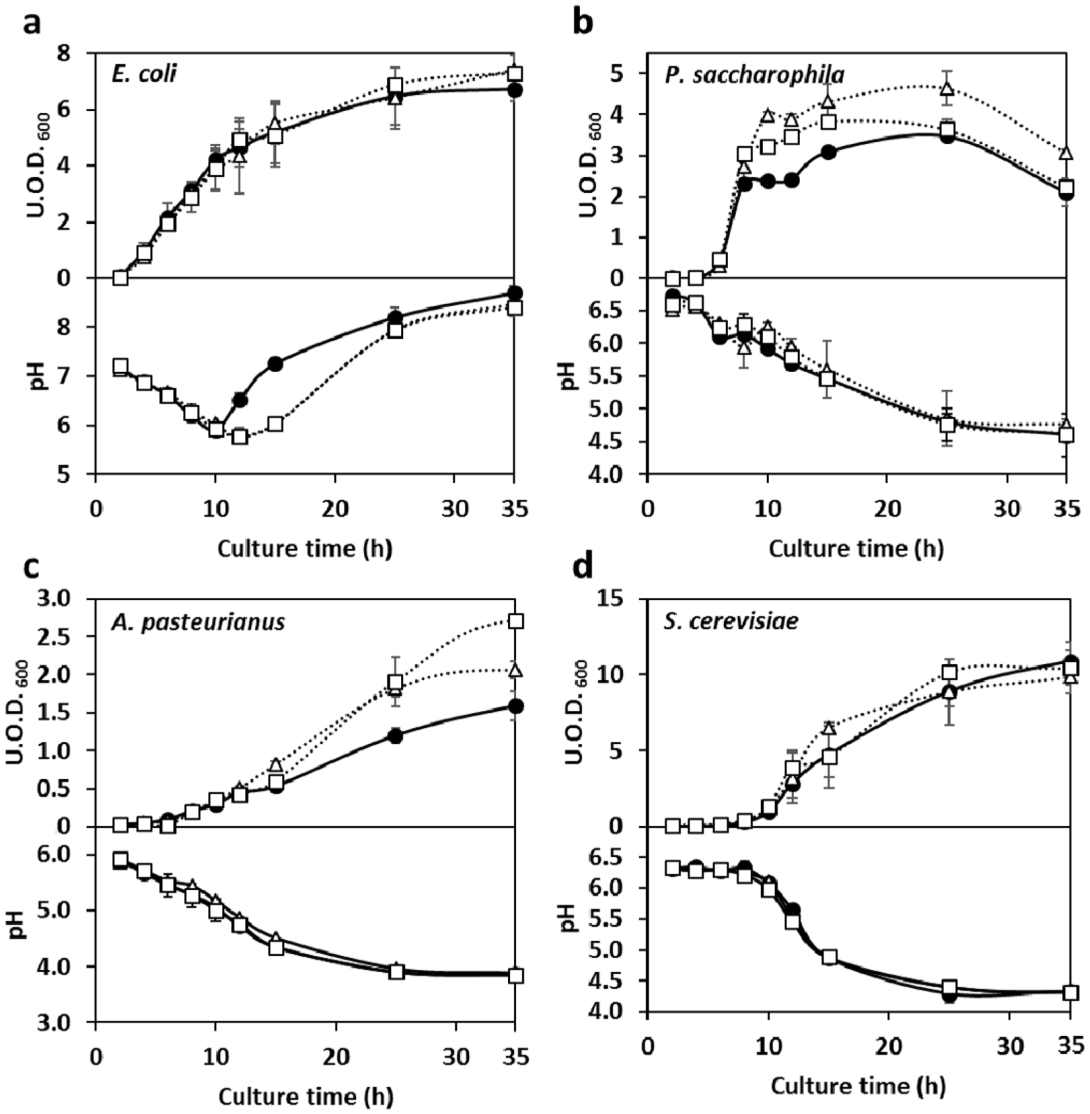
Influence of intermittent CO_2_ aeration by AAFS on shaking cultures of following 4 microorganisms; *E. coli*, a; *P. saccharophila*, b; *A. pasteurianus*, c; *S. cerevisiae*, d. Filled circle, control culture; Open triangle, condition (**a)**; Open square, condition (**b)**. Conditions **a** and **b** were aerated with 99.8% (v/v) CO_2_ at 30 mL/min using the AAFS for 90 and 30 s, respectively, after sampling. Shake-flask conditions were 100 mL of LB, R2A, NBRC no. 804 or YM medium at 30 °C, 100 (*E. coli* and *P. saccharophila*) or 200 (*A. pasteurianus* and *S. cerevisiae*) rpm shaking rotation with 70 mm amplitude, and 500 mL Erlenmeyer flask capped with breathable culture-plug.

To investigate the effect of unintentional CO_2_ accumulation in shake-flask cultures of *Escherichia coli* and *Pelomonas saccharophila*, each time sampling was performed without interruption of shaking and then 99.8% (v/v) CO_2_ was aseptically aerated into the gas phase of the flask for 90 and 30 s at 30 mL/min using AAFS. This sampling and intermittent aeration occurred after 2, 4, 6, 8, 10, 12, 15, 25, and 35 h of culturing. Compared with that in the corresponding control cultures (without aeration), the pH was different in the *E. coli* cultures, while the U.O.D._600_ was different in the *P. saccharophila* cultures **(**Fig. 6a,b**)**. Intermittent aeration of *E. coli* cultures with CO_2_ by AAFS had no effect on the U.O.D._600_ time course **(**Fig. 6a**)**. However, the pH of the *E. coli* culture decreased similarly under all the conditions until 10 h. The pH of the control culture increased sharply between 10 and 15 h, but for conditions **a** and **b** (90 and 30 s aeration, respectively), the pH increase occurred between 15 and 25 h. The pH values between 25 and 35 h were almost the same under all conditions **(**Fig. 6a**)**.

Intermittent aeration of *P. saccharophila* cultures with CO_2_ by AAFS had no effect on the pH time course. Under all conditions, the pH decreased gradually to ~4.5 **(**Fig. 6b**)**. The U.O.D._600_ of *P. saccharophila* cultured under **a** and **b** conditions (90 and 30 s aeration, respectively) gradually increased up to 6 h, similar to that of the control **(**Fig. 6b**)**. Condition **b** produced a U.O.D._600_ value that was higher than that of the control between 8 and 15 h (considered the stationary phase), which subsequently converged with the control value **(**Fig. 6b**, square)**. In the case of condition **a**, the growth phase was prolonged by 2 h compared to that of the control; the U.O.D._600_ rose at ~10 h, and then a slight decrease was observed **(**Fig. 6b**, triangle)**. However, the U.O.D._600_ remained higher than in the other conditions (control and **a**). The U.O.D._600_ values at 10 h were approximately 2.38 (control), 3.22 (condition **b**), and 4.00 (condition **a**).Thus, the U.O.D._600_ obtained with condition **a** was 1.68 times higher than that of the control. In the dead phase (35 h), the U.O.D._600_ values were 2.10 (control), 2.22 (condition **b**), and 3.06 (condition **a**), and the value with condition **a** was 1.46 times higher than that of the control. The same culture experiments were performed using *A. pasteurianus* and *S. cerevisiae* as aerobic microorganisms **(**Fig. 6c,d**)**. As compared with the control without CO_2_ aeration by AAFS, there was almost no change in pH change during the time course of both *A. pasteurianus* and *S. cerevisiae* **(**Fig. 6c,d**)**. In the case of U.O.D._600_, *A. pasteurianus* marked differences, but *S. cerevisiae* showed little change. The U.O.D._600_ of *A. pasteurianus* cultured under **a** and **b** conditions (90 and 30 s aeration, respectively) gradually increased up to 15 h, similar to that of the control. Thereafter, the values of U.O.D._600_ under these conditions (**a** and **b)** rapidly increased until 35 h and reached the stationary phase. The control continued to increase slowly until 55 h. The U.O.D._600_ values at 35 h were approximately 1.59 (control), 2.06 (condition **a**), and 2.71 (condition **b**) **(**Fig. 6c**)**. Thus, the U.O.D._600_ obtained with condition **b** was 1.7 times higher than that of the control.

## Discussion

Conventional devices for direct monitoring of flask cultures involve attaching a sensor and a detector to the inner surface of the flask^4^. These devices typically cannot withstand the very high temperatures of combustion. Even if they could, the distribution of combustion gas in the gas phase of the flask is not uniform, as CFD analysis has shown, and it is difficult to obtain reproducible data by direct measurement. However, the bypass type of CDMSS can be used to monitor CO_2_ concentration without the influence of heat; the gas in the headspace is mixed and the monitoring site is outside the flask. In this study, we demonstrated for the first time, to the best of our knowledge, that flame sterilization with a Bunsen burner during standard sampling of shake-flask cultures generates CO_2_ due to combustion and that high concentrations of CO_2_ accumulate in the headspace of the flasks. In the CDMSS technique, the gases in the flask gas phase are intentionally mixed; thus, the actual gas distribution is not assessed and the inflow of fresh air from the flask port cannot be ignored. Therefore, the flame sterilization procedure during sampling is filling the flask gas phase with CO_2_ concentrations that are higher than the actual value. This view is supported by CFD analysis, which confirmed that combustion gas with a CO_2_ concentration (~9% (v/v) maximum) higher than the value measured by CDMSS, was flowing into the headspace of the flask.

As the flame sterilization time shortened, the CO_2_ concentration in the headspace of the flask lowered, and the influence on the culture lessened. In order to perform flame sterilization with minimal impact on the culture, it may be preferred to heat the neck located below the mouth of the flask instead of opening the mouth for a very short time. However, the Bunsen burner flame near the mouth of the flask creates a temporary negative pressure, and combustion gas containing high CO_2_ concentrations near the burner flows into the flask headspace. This phenomenon cannot be avoided during the flame sterilization operation. Flame sterilization using a Bunsen burner performs two roles: **1**, sterilizing microorganisms attached to the flask mouth and culture-plug surface due to high temperatures; **2**, blowing away attached microorganisms and dust due to the high airflow. This flame sterilization operation is a universal method used during microbial cultivation, not only for flask cultures but also for test tube slant cultures. It is also performed at the time of subculturing and inoculation. Currently, there is no method that accomplishes both points **1** and **2** above without the accumulation of CO_2_. High-speed hot air treatment can be considered as an alternative to flame sterilization. However, hot air is more difficult to see than a flame.

It has been reported that standard sampling of shake-flask cultures involving the cessation of shaking leads to sharp decreases in oxygen transfer rate during sampling^11,14^. Opening the culture-plug also leads to unplanned ventilation of the gas phase in the flask^15^. The present study has shown that the flask gas phase is altered by the flame sterilization operation. In order to perform shake-flask culturing with good reproducibility, it is necessary to prepare the flasks for sampling^5^ and standardize the conventional sampling operations. Furthermore, it is necessary to construct a system that permits sampling without interrupting the shaking. Sampling techniques for built-in bioreactors, which cannot be transferred to clean benches, have been established, and by applying the same techniques to shake flasks, new sampling methods are expected to be developed in the future.

Balancing pH and CO_2_ in culture experiments is very critical. When dissolved in culture medium, CO_2_ exists as three forms: CO_3_^2−^, HCO_3_^−^, and CO_2_. In shaking cultures of *E. coli*, the pH decreased during the first 10 h, but then increased; thus, the pH fluctuated between 5.8 and 8.7. In the pH range of 5.8 to 6.8, CO_3_^2−^ is hardly present and carbon dioxide exists mainly as HCO_3_^−^ and CO_2_^17^. In particular, when the pH is higher than ~6.3, HCO_3_^−^ becomes the main component and tends to buffer the pH. Accordingly, when CO_2_ was intermittently aerated by AAFS, the pH was maintained at ~6 within the first 10 h and was comparable to the value obtained in the control cultures (no aeration).The growth of *E. coli* was minimally affected because the pH was within its growth range^18^. It was suggested that the same applies to *S. cerevisiae* whose growth was not changed by intermittent CO_2_ aeration. Regarding the growth inhibition of *S. cerevisiae*, it was reported that the CO_2_ concentration did not change up to 50% in the culture using the reactor^19^. In the shake-flask culture of *P. saccharophila*, the pH gradually decreased from 6.7 at the start of culturing to 4.6 at the end. Even in this pH range, the carbon dioxide existed mainly as HCO_3_^−^ and CO_2_ because CO_3_^2−^ is mainly present under strongly alkaline conditions. This culture was more acidic than the *E. coli* culture, and especially below pH 6.3, CO_2_ is the main component. Certainly, the pH fluctuation was smaller than that of the control, although it was only slight (around pH 6) during the first 6–10 h of culture. After 12 h of culture, buffering by CO_2_ aeration was not observed, but growth under intermittent CO_2_ aeration was improved compared with that of the controls. This was probably because the dissolved CO_2_ concentration was maintained at high levels, and not because of pH buffering capacity. It seemed that the growth of *A. pasteurianus* increased by intermittent CO_2_ aeration like the growth of *P. saccharophila*. It should also be noted that when the surface of the flask mouth becomes cloudy during flame sterilization, acidic (pH ~ 3) condensed water is generated, containing CO_2_ and nitrogen oxides.

Intermittent fluctuations in CO_2_ concentration in the flask gas phase, caused by the flame sterilization operation, have various effects depending on the type of microorganisms during shaking culture. In this study, the microorganisms were classified into the following three categories; *E. coli*, in which the pH changes, but the growth does not change; *P. saccharophila* and *A. pasteurianus*, in which the pH does not change, but the growth increases; *S. cerevisiae*, in which both the pH and growth do not change. However, all culture conditions of all the microorganisms have not been investigated and, some microorganisms may have other characteristics. To improve the desired characteristics of microorganisms (e.g., growth and metabolite production), a detailed examination of the culture conditions, such as the culture vessel (or oxygen transfer rate)^20^ and medium composition^21,22^, is very important. Selecting highly capable microorganisms^23^ and understanding various characteristics of pure strains are also very important^24–26^. In particular, CO_2_ plays roles as an intrinsic product, essential substrate, and regulatory trigger, and it is a very important factor in the culture of microorganisms and animal cells^17^. In shake-flask cultures of microorganisms, the ability to supply O_2_ has been extensively investigated, but there has been little research on CO_2_. Recently, we found that the CO_2_ produced by microorganisms during shake culture fills the gas phase of the flask, and we reported improved growth of *E. coli* by removing only the CO_2_ in the headspace and maintaining low, dissolved-CO_2_ concentrations^5^. It was reported that there was a vertical gradient of gas concentration in the flask headspace, and the CO_2_ released from the culture broth was present at a higher concentration at the bottom of the flask than at the top^13^. In the present study, the combustion gas from the flame sterilization filled the headspace of the flask with a high CO_2_ concentration and this affected the culture. Therefore, shake-flask cultures have a large difference in gas-liquid ratio and a large volume in the gas phase compared with that of bioreactor cultures. These findings strongly suggest that understanding and controlling the headspace gas environment are very important.

In this study, we demonstrated for the first time, to the best of our knowledge, that standard sampling influences the culture through CO_2_ accumulation induced by flame sterilization. In addition, accumulation of CO_2_ in the gas phase of the flask could be used to improve culturing, depending on the type of microorganism. It has been reported that purging CO_2_ by intermittent aeration, improves the production of human-like collagen in bioreactor cultures of *E. coli*^27^. The screening system for microorganisms often involves opening the culture-plug^15^. In the future, intermittent CO_2_ aeration due to the inflow of combustion gas generated by the flame sterilization used during sampling will be a very important parameter examined in shake-flask cultures. Moreover, connecting the gas aeration unit (e.g., AAFS) and monitoring device (e.g., CDMSS) by proportional–integral–derivative (PID) controller will allow more precise control of the headspace (e.g., maintenance of constant or stepwise changes in CO_2_ and O_2_ concentrations), which will be possible with the use of flasks with breathable culture-plugs. Shake-flask culture is already advancing with the advent of fed-batch systems for pH and nutrient control^28–31^. In order to understand the reciprocity and reproducibility among various culture vessels and reactors well, both the development of special shake-flask culture systems and the comprehensive elucidation of many unknown factors such as the monitoring method and sampling operation (included inoculation) will be very important for the future of shake-flask culturing.

## Methods

### Analysis of manual flame sterilization in sampling process using CDMSS

Manual flame sterilization was performed using a Bunsen burner, 500 mL Erlenmeyer flask with small pipes on the sides, and CDMSS. During the flame sterilization process, the gas in the headspace of the flask was monitored using CDMSS by circulation at 50 mL/min, and the maximum CO_2_ concentration was recorded. The manual flame sterilization was performed for 5, 10, and 20 s; each time point was repeated four or more times, and the average value was calculated. All experiments were performed at room temperature (approximately 25 °C).

### Analysis of flame sterilization operation with fixed operating angle and time using CDMSS

The system shown in Supplementary Fig. S1 online was constructed, and the effect of flame sterilization on the CO_2_ concentration in the headspace of the flask was analyzed. Although the flask was brought near the Bunsen burner in the manual experiments, in this system, the position and angle of the flask were fixed when the Bunsen burner was brought close to the flask. After the operation time, the slide plate was moved to separate the Bunsen burner from the flask while monitoring the headspace of the flask using CDMSS **(**see Supplementary Fig. S1 online, Figs. 1–6**)**, and the maximum CO_2_ concentration was recorded. The operation time and inclination angle were set with reference to the manual flame sterilization procedure, namely 5, 10, or 20 s, and 15, 25, or 35°, respectively. All experiments were performed at room temperature in four replicates, and the average values were calculated.

### Measurement of CO_2_ concentration generated by flame sterilization procedure mimicked using CDMSS and AAFS

In order to investigate the effect of only the flame sterilization operation on the shake-flask culture without the other sampling processes, we connected a 99.8% (v/v) CO_2_ gas cylinder to the AAFS to aerate intermittently the headspace of the shaking flask^15^. The CO_2_ concentration in the gas phase of the flask was monitored using CDMSS under various intermittent aeration conditions, and the maximum CO_2_ concentration was recorded. All experiments were performed at room temperature in four replicates, and the average values were calculated.

### Computational domain and settings

Supplementary Fig. S6 online shows the schematics of the computational domain. A Bunsen burner, with a pipe size of 12.7 mm for its outer diameter and a thickness of 1.00 mm, was installed upwards. The capacity of the Erlenmeyer flask was 500 mL, and it was empty. The lower part of the flask tip was installed 60 mm above the burner rim top and the flask was tilted 60 °. The X, Y, and Z directional sizes of the computational domain were 450 mm, 320 mm, and 370 mm, respectively, and the domain was divided into 920,000 cells.

Calculations were conducted by using Fluent 2019R1 (ANSYS Inc., Canonsburg, U.S.A.). The physical modeling was as follows. The transport equations of mass, momentum, energy, and species were solved as laminar flow, and CH_4_, O_2_, CO, CO_2_, H_2_O, and N_2_ were considered chemical species, with their thermal properties depending on temperature. Westbrook and Dryer’s two-step reaction mechanism was employed^32^. Gravity was also considered. The time step was 5 × 10^−4^ s and the maximum iteration number per time step was 20. The calculation was continued until the flow time reached 20 s.

As boundary conditions, the inlet flow rate of the Bunsen burner was 6.53 × 10^−5^ m^3^/s at standard state. The fuel was CH_4_ and the oxidizer was air, which consisted of 21.0% O_2_ and 79.0% N_2_. The equivalence ratio *Φ* of the premixed gas was 2.00. Here, the equivalence ratio is defined as $\varPhi =\frac{\frac{{X}_{CH4}}{{X}_{O2}}}{{\left(\frac{{X}_{CH4}}{{X}_{O2}}\right)}_{st}}$, where *X*_*i*_ is the mole fraction of species *i*, the subscript *st* denotes stoichiometric conditions, and ${(\frac{{X}_{CH4}}{{X}_{O2}})}_{st}$ is 0.5. For the remaining lower interface, 0.100 m/s was set as the upward ambient co-flow; the component was air. The temperature of these gases was 26.85 °C. The surrounding boundaries other than the lower parts were set as pressure outlets, and when inflowing from outside, the gases were air at 26.85 °C. The flask and burner were made of borosilicate glass and 304 stainless steel, respectively. For the borosilicate glass, the density, heat capacity, and thermal conductivity were 2,190 kg/m^3^, 740 J/kg-K, and 1.38 W/m-K, respectively. The corresponding values for the stainless steel were 7,930 kg/m^3^, 590 J/kg-K, and 16.7 W/m-K, respectively.

In the simulation process, a premixed flame was first formed without installation of the flask. After the flask was positioned, the temperature of the flask and inside the flask was set as ambient temperature and the inside filled with air. This time was defined as *t* = 0 s and a transient calculation was conducted.

### Empirical verification

A simple experiment was also conducted for verifying the accuracy of the simulation. The sizes and positions of the burner and flask were the same as those in the simulation in the previous section. The fuel was CH_4_ with a purity of > 99.5% and the oxidizer was ambient air. The flow rate of the fuel was controlled by mass flow meter, which was 0.680 L/min at standard state.

Supplementary Fig. S7 online compares the flame shapes between CH_4_ distributions in the simulation and a direct photograph of the experiment, without the flask. The flame shape in the simulation qualitatively corresponds with that in the experiment. Two flames, an inner and an outer flame, are formed. The height of the inner flame is approximately 40 mm. For the outer flame, although the height cannot be accurately measured because its tip is unclear, it is presumed to be approximately 80 mm. Supplementary Fig. S8 online shows the experimental setup for heating the flask. The flask was suspended by thin wires. Four K-type sheathed thermocouples (1.0 mm in diameter) were installed inside the flask. In order to measure the surface temperature of the flask using a non-contact infrared thermography device, the flask was colored black. Supplementary Fig. S9 online shows the temperature along the center axis of the flask at *t* = 20 s. In the experiment, these temperatures were measured at heights of 42, 72, 102, and 142 mm (see Supplementary Fig. S9 online, closed red circle). The temperatures for both the simulation and experiment were ambient from 0–100 mm. At > 120 mm, the temperature drastically increased in the simulation, approaching 1,400 °C at 172 mm. In contrast, in the experimental result, the temperature at 142 mm was 75.2 °C, which was 130 °C lower than the corresponding simulation. There are two main reasons that the temperature in the simulation was higher. First, a two-step reaction mechanism was used. For *Φ* = 2.0, the adiabatic flame temperature was 270 °C higher than the value when employing detailed mechanisms. Second, the dilution of burned gas by co-flows was different from that occurring in a real situation. Here, 0.1 m/s was set as the co-flow velocity because a calm flow field was assumed; flow fields might be more turbulent in the laboratory. In addition, according to the infrared thermography device, the maximum temperature of the flask exceeded 500 °C at *t* = 180 s, but the area where the temperature was > 100 °C was limited to the neck regions with heights > 130 mm.

As indicated above, we confirmed that our simulation can qualitatively evaluate the behavior of burned gas around the flask.

### Microorganisms and medium

*E. coli* K12 IFO3301, *P. saccharophila* NBRC103037, *A. pasteurianus* NBRC3283, and *S. cerevisiae* IFO0309 were used in this study. The LB medium used for *E. coli* cultivation consisted of (g/L): tryptone, 10; yeast extract, 5; and NaCl, 5. The R2A medium used for *P. saccharophila* cultivation consisted of (g/L): yeast extract, 0.5; peptone, 0.5; casamino acids, 0.5; glucose, 0.5; soluble starch, 0.5; sodium pyruvate, 0.3; K_2_HPO_4_, 0.3; and MgSO_4_·7H_2_O, 0.05. The NBRC no. 804 medium used for *A. pasteurianus* cultivation consisted of (g/L): hipolypepton, 5; yeast extract, 5; _D_-glucose, 5; MgSO_4_·7H_2_O, 1. The YM medium used for *S. cerevisiae* cultivation consisted of (g/L): _D_-glucose, 10; peptone, 5; yeast extract, 3; and malt extract, 3.

### Inoculum preparation

A loop-full of *E. coli* IFO3301, *P. saccharophila* NBRC103037, *A. pasteurianus* NBRC3283 or *S. cerevisiae* IFO0309 slant culture was inoculated into a 500 mL Erlenmeyer flask containing 100 mL of LB, R2A, NBRC no. 804 or YM media, respectively. The samples were then cultivated at 30 °C on a rotary shaker with a 70-mm shaking diameter at 100 rpm for 6.75 h (*E. coli*) or 20 h (*P. saccharophila*) and 200 rpm for 72 h (*A. pasteurianus*) or 14 h (*S. cerevisiae*). Glycerol stocks were prepared by adding the culture medium to glycerol (final concentration 20% [v/v]) and stored at −80 °C.

### Culture conditions

One millilitre of each glycerol stock was inoculated into a 500 mL Erlenmeyer flask containing 100 mL of 4 media types and cultured at 30 °C on a rotary shaker with a 70-mm shaking diameter at 100 rpm (*E. coli* and *P. saccharophila*) or 200 rpm (*A. pasteurianus* and *S. cerevisiae*). For air permeability, the Erlenmeyer flask was equipped with a BIO-SILICO N-38 sponge plug (Shin-Etsu Polymer Co., Ltd, Tokyo, Japan; breathable culture-plug). After each sampling of the culture broth, 99.8% (v/v) CO_2_ gas was aseptically aerated into each flask headspace using the AAFS for either 90 s or 30 s at 30 mL/min.

### Sampling of culture broth during shake-flask culture

Conventional sampling methods, which include interruption of shaking, opening the culture-plug, and flame sterilization on a clean bench, are known to affect the cultured microorganisms. Therefore, sampling during the shake-flask culturing was performed with the system shown in Supplementary Fig. S10 online, which is similar to the CDMSS sampling method^5^. Specifically, the sampling unit and flask were autoclaved independently. Then, the sampling unit, flask, three-way plug, and 50 mL syringe with a 0.2 *μ*m filter were assembled on a clean bench, as shown in Supplementary Fig. S10 online. The three-way plug, 50 mL syringe, and 0.2 *μ*m filter were disposable. Sampling of the shake-flask culture was performed by attaching a syringe to a three-way plug connected to a sampling tube. After collecting the culture broth, in order to ensure its removal from the sampling tube, a volume of fresh air (0.2 *μ*m filtered) was used to flush the broth out of the tube. The sampled culture broth was stored at −80 °C until used for analysis.

### Measurement of culture factors

The U.O.D._600_ and pH of the culture broth, which was sampled without interruption of shaking, were measured using a V-570 spectrophotometer (JASCO, Tokyo, Japan) and a pH meter (HORIBA, Kyoto, Japan), respectively. In order to minimize the decrease in volume of the culture broth due to repeated sampling, the total sampled volume did not exceed 10% (v/v) of the total volume of the initial culture medium. All measurements were performed in duplicate. In the case of *P. saccharophila*, the culture broth contained cell aggregates; these aggregates were disrupted by vortex and ultrasonication for 10 min at 4 °C prior to absorbance measurements.

## Supplementary information

Supplementary information.
