# Supplementary material for: Analysis of the influence of flame sterilization included in sampling operations on shake-flask cultures of microorganisms
Source: Sci Rep. 2020 Jun 30;10:10385. doi: 10.1038/s41598-020-66810-3 (PMC7326993; doi:10.1038/s41598-020-66810-3)
Supplement: Supplementary file 1 — Supplementary information. [file 41598_2020_66810_MOESM1_ESM.docx]

Title

**Analysis of the influence of flame sterilization included in sampling operations on shake-flask cultures of microorganisms**

Authors:

Masato Takahashi^1^, Takafumi Honzawa^2^, Ryuichi Tominaga^2^, Hideki Aoyagi^1^*

Address:

^1^Faculty of Life and Environmental Sciences, University of Tsukuba, Tsukuba, Ibaraki 305-8572, Japan

^2^Combustion of Thermo and Fluid Dynamics, Department of Fundamental Technology, Tokyo Gas Co. Ltd., Yokohama, Kanagawa 230-0045, Japan

*Corresponding author:

Hideki Aoyagi

Tel.: +81 29 853 7212

Fax: +81 29 853 4605.

E-mail: aoyagi.hideki.ge@u.tsukuba.ac.jp

**Supplementary Information**

**
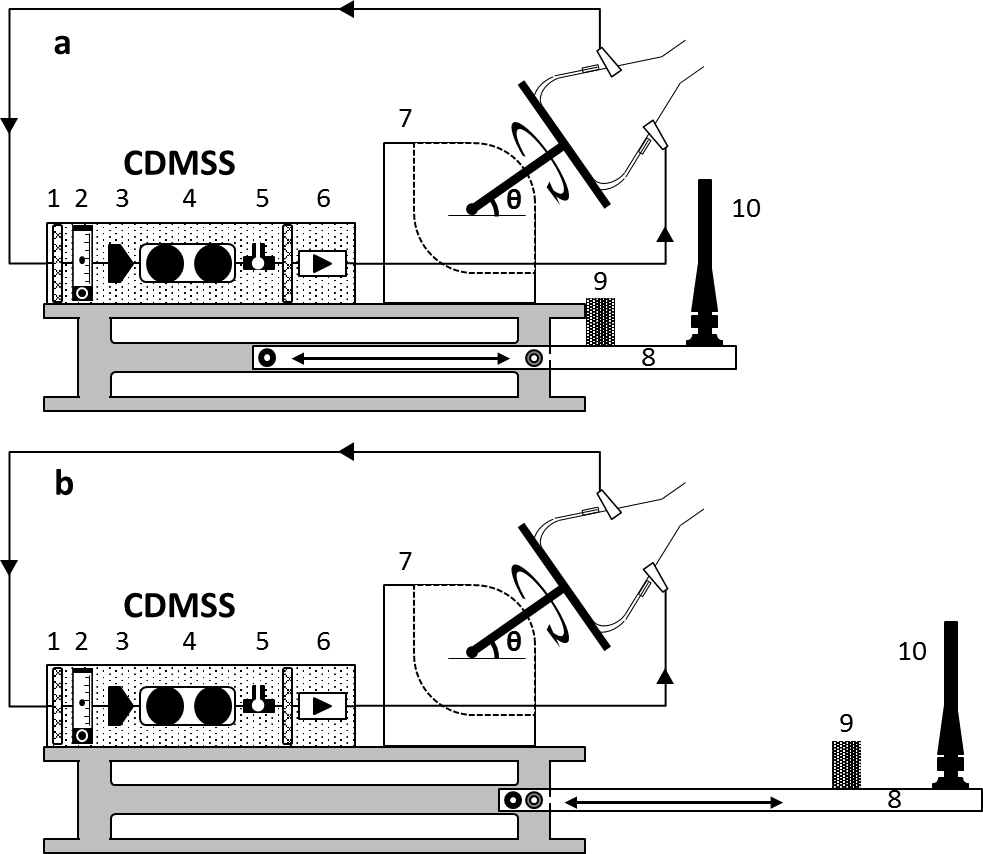
**

**Fig. S1** Conceptual diagram of analysis of flask headspace in simulated manual flame sterilization operation.

Components: **1**, 0.22 μm filter; **2**, flow meter; **3**, diaphragm pump; **4**, gaseous measuring unit; **5**, sampling unit; **6**, check valve; **7**, flask fixture; **8**, slide table with fixed burner; **9**, stopper; **10**, Bunsen burner; θ, flask inclination angle. The component parts of CDMSS are **1** – **6**. During flame sterilization, slide **8** is moved into position **a**; when the operation is finished, slide **8** is moved into position **b**.

**
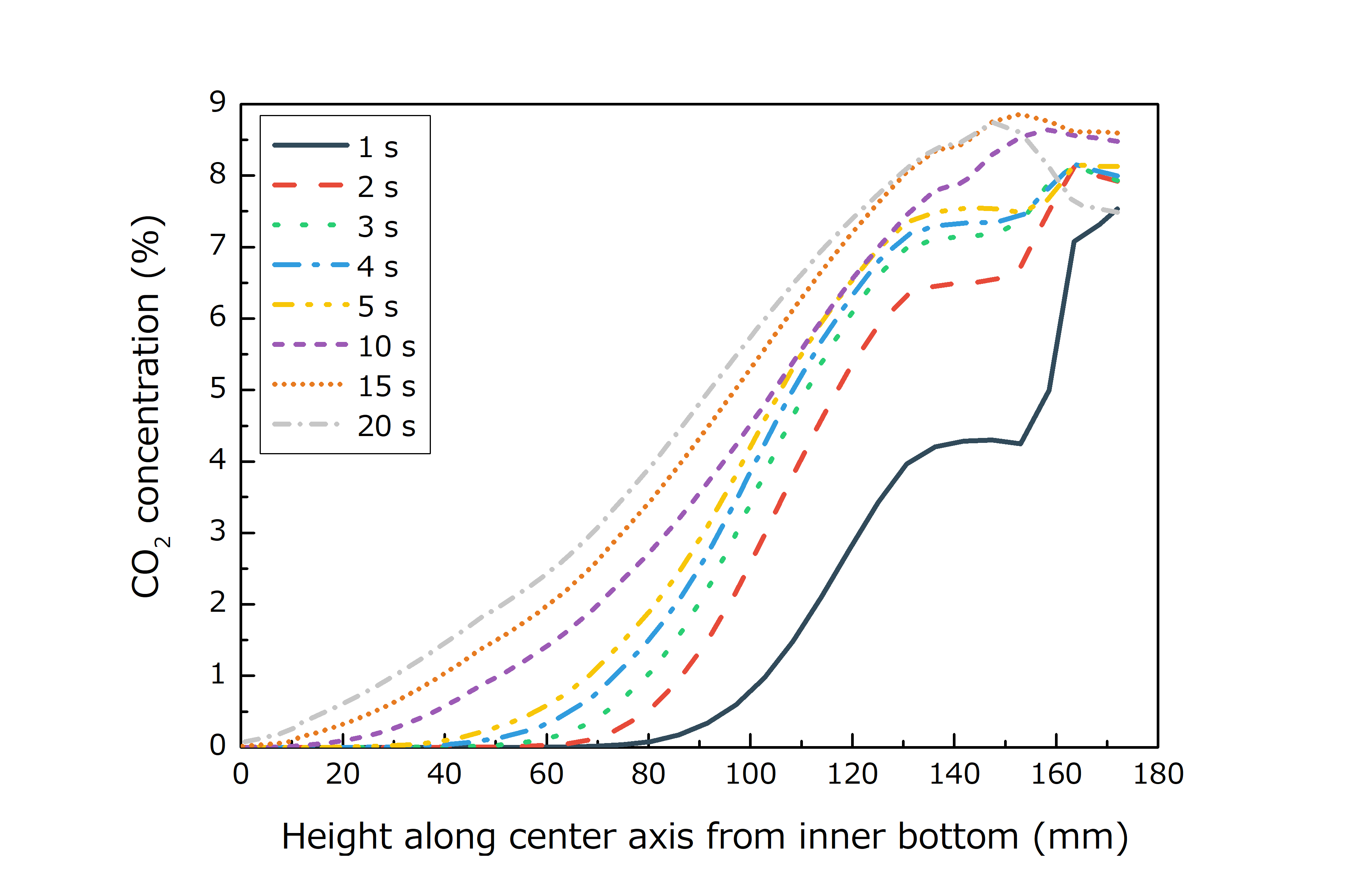
**

**Fig. S2** Time series change in CO_2_ distribution.


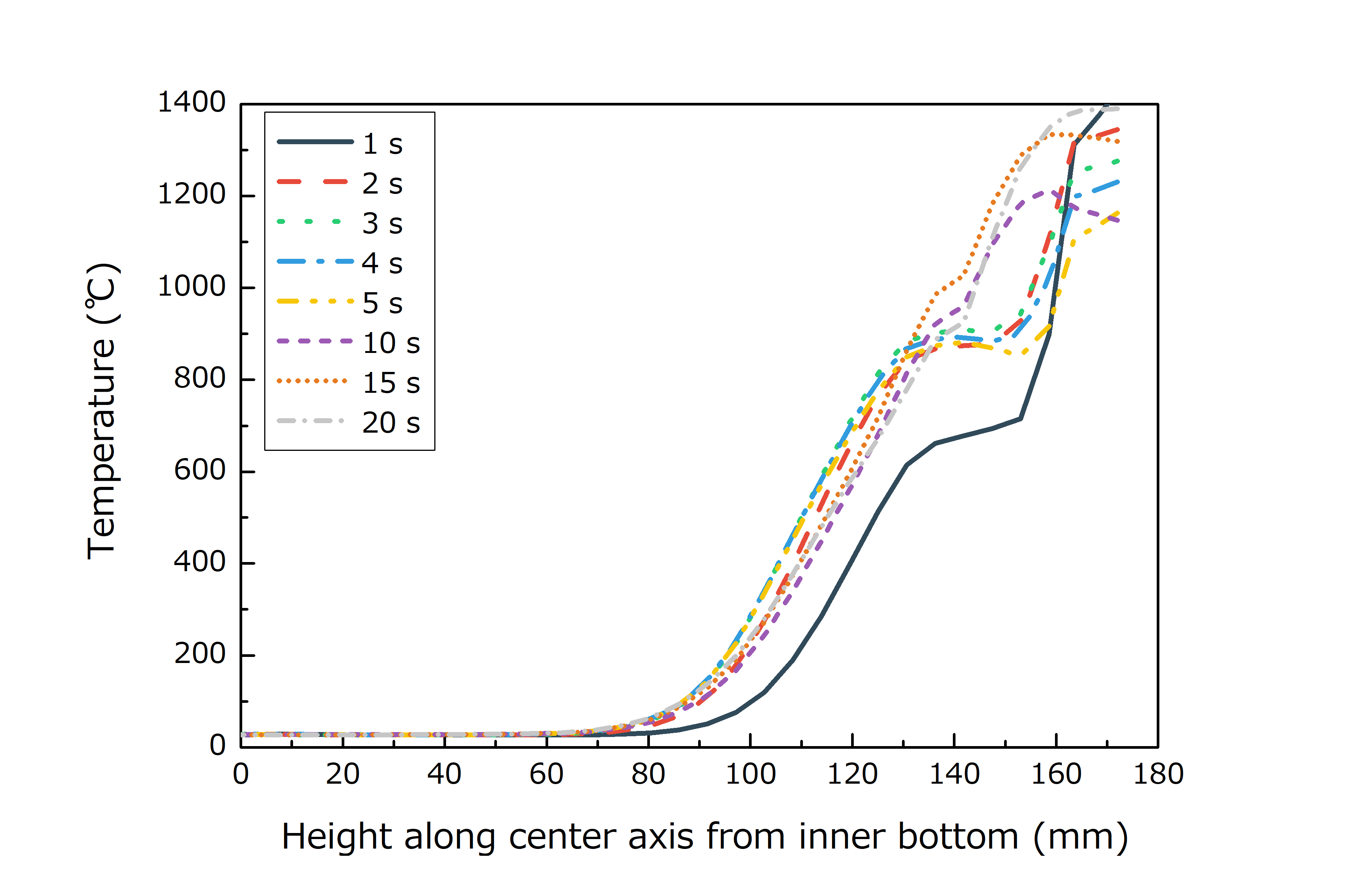


**Fig. S3** Time series change in temperature distribution.

**
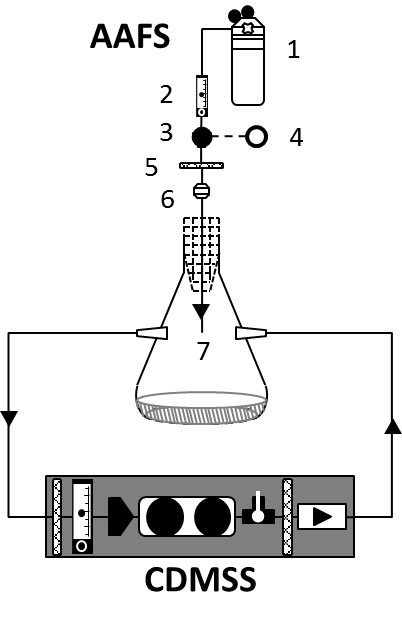
**

**Fig. S4** Conceptual diagram of experimental setup using AAFS, mimicking the accumulation of CO_2_ caused by flame sterilization.

Components: **1**, 99.8% (v/v) CO_2_ cylinder; **2**, flow meter; **3**, solenoid valve; **4**, control system; **5**, filter; **6**, connector; **7**, needle. Grey shading indicates CDMSS.

**
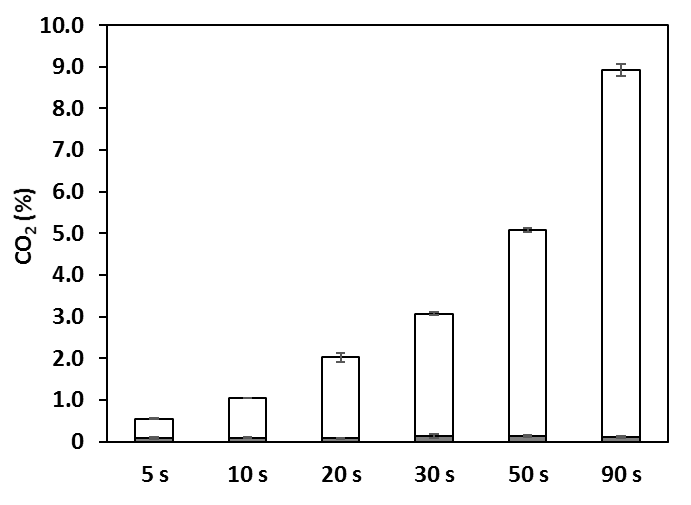
**

**Fig. S5** CO_2_ accumulation in the flask headspace caused by flame sterilization operation using AAFS

White bars, maximum concentration; Grey bars, initial concentration. Error bars indicate standard deviations (n  =  4).

**
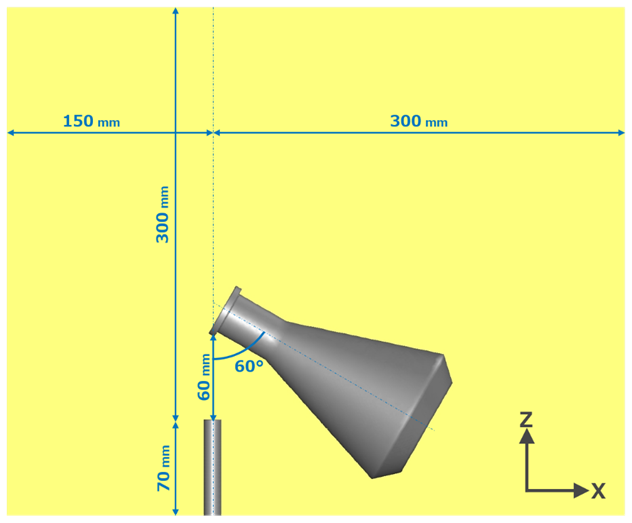
**

**Fig. S6** Schematics of computational domain.

**
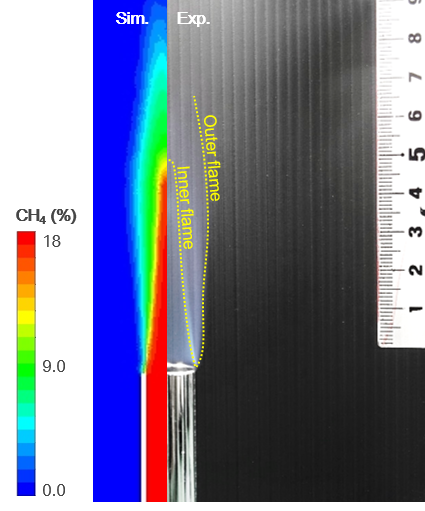
**

**Fig. S7** Comparison of flame shapes between methane distributions with theoretical (simulation, left side) and empirical (photograph, right side) data.

**
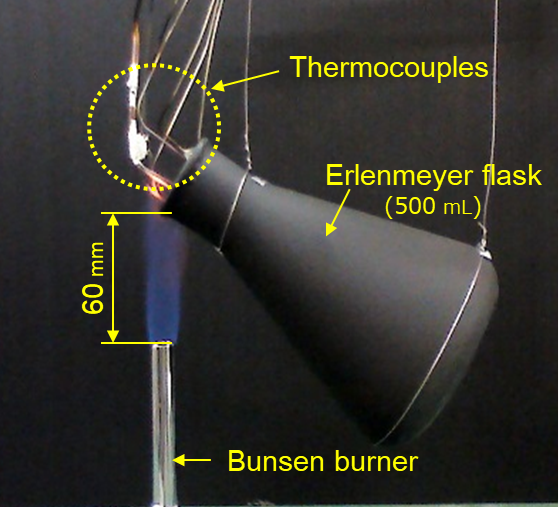
**

**Fig. S8** Experiment for heating flask.

**
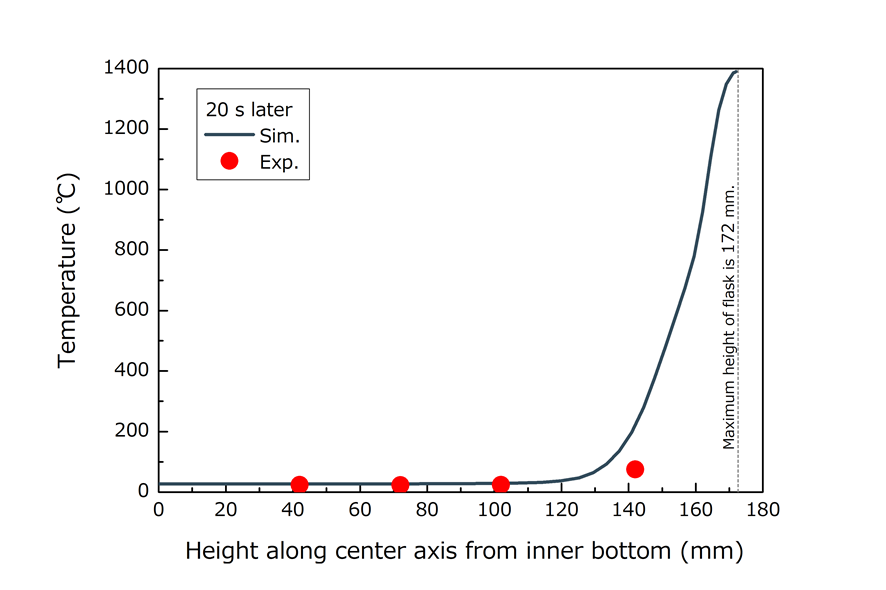
**

**Fig. S9** Temperature along centre axis of flask at t = 20 s.

**
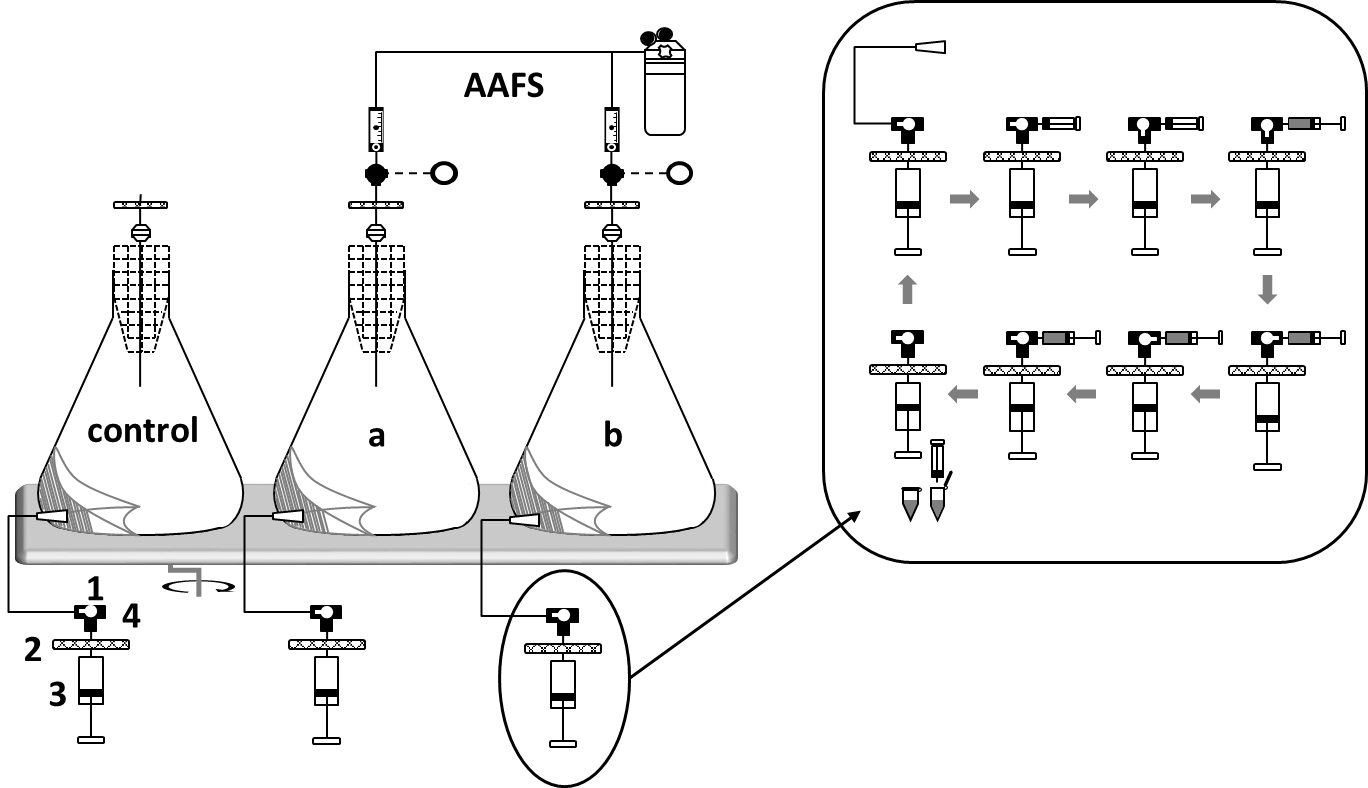
**

**Fig. S10** Conceptual diagram of the sampling method without shaking interruption for the shake-flask cultures of *E. coli*, *P. saccharophila*, *A. pasteurianus* and *S. cerevisiae*.

Conditions **a** and **b** represent aeration of 99.8% CO_2_ into the headspace by AAFS for 90 and 30 s after sampling, respectively. The **control** is not aerated, but connected to a part of AAFS to perform the comparative experiment. The box shows the sampling procedure used in this study. Components: **1**, sampling unit; **2**, 0.22 μm filter; **3**, syringe; **4**, sampling port.
